# Shared molecular mechanisms between type 2 diabetes and thyroid cancer: integrated bioinformatics insights for prognostic biomarker discovery

**DOI:** 10.1530/EC-25-0181

**Published:** 2025-10-06

**Authors:** Jiahui Qi, Chuanzhi Chen, Feng Zhu, Chuankai Chen, Yue Wang

**Affiliations:** ^1^ Institute of Aging, Key Laboratory of Alzheimer’s Disease of Zhejiang Province, Wenzhou Medical University, Wenzhou, Zhejiang, China; ^2^ Department of Thyroid Surgery, National Key Clinical Specialty (General Surgery), The First Affiliated Hospital of Wenzhou Medical University, Wenzhou, Zhejiang, China; ^3^ Department of Child Healthcare, The Third Clinical Institute Affiliated to Wenzhou Medical University, Wenzhou, Zhejiang, China; ^4^ Department of Emergency Medicine, The First Affiliated Hospital of Wenzhou Medical University, Wenzhou, Zhejiang, China; ^5^ Department of Metebolic Surgery, Shanghai Tenth People’s Hospital, Tongji University School of Medicine, Shanghai, China

**Keywords:** thyroid cancer, type 2 diabetes mellitus, bioinformatics, prognostic biomarkers, insulin resistance

## Abstract

**Background:**

Thyroid cancer (TC) is a prevalent endocrine malignancy with rising global incidence, particularly among women. Emerging evidence suggests a significant association between type 2 diabetes mellitus (T2D) and TC, potentially mediated by hyperinsulinemia, insulin resistance, and chronic inflammation. However, the molecular mechanisms linking these diseases remain poorly understood.

**Methods:**

We integrated transcriptomic datasets from the Gene Expression Omnibus (GEO) database (GSE33630, GSE35570, GSE60542 for TC; GSE86468 for T2D) to identify shared differentially expressed genes (DEGs). Functional enrichment, protein–protein interaction networks, and Cox regression analyses were employed to elucidate pathways and prognostic biomarkers.

**Results:**

We identified 28 shared DEGs between TC and T2D, with CD44, TGFBI, RUNX2, and GJA1 as key hub genes. Kyoto Encyclopedia of Genes and Genomes (KEGG) analysis highlighted pathways involving cell adhesion, extracellular matrix remodeling, and NF-κB signaling. A risk model incorporating seven genes (e.g., PRDM1 [protective] and ZFPM2 [risk]) stratified TC patients into high- and low-risk groups with distinct survival outcomes (*P* = 0.017).

**Conclusion:**

T2D and TC exhibit overlapping genetic dysregulation, particularly in pathways governing metabolic reprogramming and tumor microenvironment crosstalk. Notably, PRDM1 and ZFPM2 may serve as therapeutic targets for TC in patients with concurrent diabetes.

## Introduction

Thyroid cancer (TC) is one of the most common malignancies of the endocrine system worldwide, with a marked female predominance. In 2022, there were 821,000 new cases of TC and 47,000 deaths worldwide, accounting for 4.1% of all cancer cases and 0.5% of cancer-related deaths (1). The standardized incidence rate of TC in women is approximately three times higher than that in men, while the standardized mortality rates between men and women are similar. East Asia exhibits the highest global incidence of TC, a phenomenon influenced by a multifactorial interplay of genetics, lifestyle, environmental pollution, hormonal levels, and radiation exposure (2). Furthermore, recent studies have indicated that metabolic disorders, such as obesity and thyroid dysfunction, may increase the risk of developing TC (3). With the continuous advancements in early screening and diagnostic techniques, an increasing number of TC cases are being detected and treated promptly. However, despite the generally favorable prognosis for most TC cases, treatment remains challenging for more aggressive types, such as anaplastic TC and medullary TC (4).

There is a notable association between T2D and TC. Several studies have shown that individuals with diabetes are more likely to develop TC, particularly papillary thyroid cancer (PTC), compared to non-diabetic (ND) individuals. Specifically, hyperinsulinemia and insulin resistance may promote the proliferation and growth of TC cells by activating the insulin-like growth factor (IGF) pathway (5). IGF-1 is considered a key factor closely associated with the development of several cancers, including TC (6). Furthermore, hyperglycemia and chronic inflammation in T2D patients are also believed to be significant contributing factors to TC development (7). According to a large cohort study, T2D patients are at a notably higher risk of TC than ND individuals, with this risk being particularly evident in men (8). The relationship between T2D and TC extends beyond cancer occurrence, influencing both the treatment process and prognosis of diabetic patients. One study found that thyroid hormones regulate food intake, glucose, and lipid metabolism both centrally and peripherally, involving target tissues such as the liver, white and brown adipose tissue, pancreatic β cells, and skeletal muscle. This mechanism helps explain the connection between subclinical hypothyroidism, T2D, and metabolic syndrome (9). These findings suggest that T2D may promote the onset and progression of TC through multiple mechanisms. Further mechanistic studies are warranted to delineate the specific molecular mechanisms linking diabetes to TC and to identify potential therapeutic strategies.

T2D has been implicated in an elevated risk of various malignancies, including pancreatic, colorectal, endometrial, hepatic, gastric, and breast cancers (10, 11, 12, 13, 14, 15). Conversely, a reduced incidence of prostate cancer has been observed among diabetic populations (16, 17). The underlying mechanisms are multifaceted, encompassing hyperinsulinemia, hyperglycemia, obesity, and chronic inflammation. These metabolic disturbances may facilitate carcinogenesis by promoting cellular proliferation and inhibiting apoptosis. Understanding the association between T2D and various cancers underscores the importance of vigilant cancer screening and preventive strategies in diabetic patients. Further investigation into the relationship between T2D and TC is warranted.

The pathophysiological interplay between T2D and TC may involve multiple interconnected mechanisms. Elevated IGF-1 levels in diabetic patients activate transmembrane IGF-1 receptors on thyrocytes, triggering the PI3K/AKT/mTOR oncogenic signaling cascade that potentiates neoplastic proliferation and invasive behavior (18, 19, 20). Notably, pharmacovigilance data suggest glucagon-like peptide 1 (GLP-1) receptor agonist therapy in T2D associates with elevated risks of differentiated and medullary TC, particularly during the initial 1–3 therapeutic years (21). Concurrently, chronic hyperglycemia drives metabolic reprogramming in TC through preferential reliance on aerobic glycolysis (Warburg effect) to fuel unrestrained growth (22, 23), while diabetes-induced mitochondrial oxidative stress generates genotoxic reactive oxygen species that impair DNA repair fidelity in thyrocytes, predisposing to carcinogenic mutagenesis (24, 25). Furthermore, thyroidal iodine concentrations modulate tumor progression through dual regulation of hypoxia-inducible factor-1α (HIF-1α) stabilization and VEGF-dependent angiogenic pathways, creating a permissive microenvironment for malignant transformation (26, 27). These multilayered mechanisms collectively underscore the metabolic-epigenetic crosstalk underlying diabetes-associated thyroid oncogenesis.

Importantly, emerging evidence suggests that an elevated preoperative glucose-to-lymphocyte ratio serves as an independent predictor of central lymph node metastasis (CLNM) in patients with T2D and PTC (28). This finding implies that hyperglycemic patients may harbor an increased risk of lymph node metastasis in TC, though the underlying mechanisms remain elusive. Furthermore, elevated fasting serum glucose (FSG) levels exhibit a significant association with progressive lymph node metastasis (LNM), particularly CLNM and combined CLNM-lateral LNM, positioning FSG as an independent prognostic marker for advanced nodal involvement. These observations underscore its potential utility in guiding lymph node dissection strategies (29). In addition, T2D correlates strongly with aggressive clinicopathological features of PTC, including higher rates of extrathyroidal extension and lymph node metastasis. Intriguingly, acarbose therapy demonstrates a protective effect against metastatic progression compared to other glucose-lowering agents, suggesting a distinct pathophysiological interplay between antidiabetic regimens and TC biology (30). Postprandial 3 h glucose metabolic parameters further correlate with increased metastatic lymph node burden (31).

In summary, robust evidence supports a complex and partially overlapping etiological relationship between T2D and thyroid carcinogenesis. Nevertheless, the exact mechanistic basis underlying this association remains incompletely characterized, necessitating systematic investigation. Moreover, a notable gap persists in bioinformatics research exploring the T2D-TC nexus. This study seeks to unravel the potential links between TC and T2D, as deciphering their interplay may yield critical insights into disease mechanisms. Leveraging a systematic bioinformatics approach, we analyze genomic data from affected tissues to dissect the nature of the T2D-TC relationship. This study pursues dual objectives: i) to identify aberrantly expressed proteins or genes in T2D that may drive thyroid oncogenesis, and ii) to elucidate their functional roles, thereby facilitating the development of targeted therapeutics. Ultimately, this work provides a foundational framework for drug discovery and precision medicine in T2D-associated TC.

## Materials and methods

### Datasets used for this research

We collected datasets from the GEO database from 2010 to the present. The datasets include both tumor and normal tissue samples, with some also featuring samples from individuals with T2D and their respective controls. We excluded datasets with fewer samples and also filtered out those that differed in data type or species. RNA sequencing data were processed in raw read counts and FPKM (fragments per kilobase million) formats. Datasets with missing or outlier values, or those that could not be properly normalized were excluded. The final datasets selected for analysis focus on PTC, including GSE33630 (49 TC samples and 45 normal thyroid samples), GSE35570 (33 pediatric and young adult PTC samples and 32 controls), and GSE60542 (28 PTC samples and 24 normal thyroid samples). The T2D dataset (GSE86468) contains gene expression data from human islets, including 24 human islet cell samples (six T2D cases and six controls) profiled using dRNA-seq.

### Cross-disease differentially expressed genes (DEGs) identification and intersection analysis

We performed differential expression analysis utilizing gene expression microarray data retrieved from the National Center for Biotechnology Information (NCBI) GEO database. Transcriptional-level differential expression refers to genes demonstrating statistically significant expression disparities under distinct experimental conditions. To identify pathology-associated DEGs, transcriptomic profiles were compared between healthy tissues and thyroid carcinoma specimens within GEO datasets, as well as between T2D and healthy control cohorts. The primary objective of this study was to extract thyroid carcinoma-related DEGs from the GSE33630, GSE35570, and GSE60542 datasets, while T2D-associated DEGs were derived from the GSE8646 dataset. Statistically significant DEGs were filtered using stringent thresholds: an absolute log fold change ≥1.0 and an adjusted *P*-value <0.05. Subsequently, Venn diagram analysis was employed to delineate overlapping DEGs between thyroid carcinoma and T2D pathophysiological processes.

### KEGG profiling of shared DEGs

Gene set expression profiling constitutes a critical bioinformatic methodology for classifying biological phenomena with shared molecular signatures, encompassing physiological processes or chromosomal loci implicated in multiple interrelated pathologies. Pathway analysis was conducted via the ‘ggkegg’ R package, enabling systematic KEGG enrichment visualization and statistical evaluation. Systematic characterization of molecular mechanisms and signal transduction pathways was conducted for common DEGs. Pathway enrichment analysis was performed on co-upregulated and co-downregulated genes shared between thyroid carcinoma and T2D, leveraging multi-species genomic databases that delineate comprehensive landscapes of gene functions, molecular interactions, and metabolic networks.

### Analysis of protein–protein interaction (PPI) networks

The PPI network, dynamically assembled upon a protein’s maturation and precise intracellular localization, serves as a functional scaffold that coordinates biological activities through collaborative macromolecular complexes. To delineate functional and structural interplay between TC and T2D, we systematically constructed a PPI network derived from common DEGs, leveraging interaction data curated from the STRING database (v11.5) (32). Multi-tiered confidence thresholds were applied to stratify interaction reliability. Highly interconnected proteins were identified through topological metrics and subsequently prioritized for functional annotation.

Hub genes, functioning as master regulators of biological networks, govern disease progression by maintaining modular architecture. Using CytoHubba’s topological analytics in Cytoscape (v3.10.2) (33), network prioritization was achieved via an integrated multi-parametric scoring system, with topological significance assessed through Bottleneck and density of maximum neighborhood component (DMNC) algorithms. This approach resolved key hubs across multilayer molecular interactions (PPI, DNA co-dependencies, and DNA-protein binding networks).

### Prognostic gene signature construction and risk stratification

The normalized expression matrix of 28 shared DEGs was subjected to median threshold-based binarization, with expression values dichotomized relative to their sample-wide median. Following integration with clinical covariates, univariate Cox proportional hazards regression (R package ‘survival’, coxph function; significance threshold *P* < 0.05) was implemented to identify prognostically relevant DEGs and establish hub genes as independent prognostic determinants. Subsequently, LASSO-penalized regression (R package ‘glmnet’) was applied to refine the prognostic signature, with parameters set to L1 regularization (*α* = 1), λ iteration count (*n*λ = 100), and significance retention criteria (*P* < 0.05). Coefficients, including the Y-intercept and hazard ratio (HR) weights for each gene, were extracted via the coef function. These metrics were integrated with hub gene expression profiles to stratify patients into distinct risk groups based on composite prognostic scores. Key candidate genes were dichotomized into high- and low-expression cohorts based on median transcriptional thresholds. To evaluate the clinical prognostic relevance of this stratification, Kaplan–Meier survival curves with log-rank testing were generated to compare inter-subgroup survival outcomes.

### Statistical analysis

All statistical analyses were conducted using R program (v4.4.0) and SPSS software (v29.0.2), with a predefined significance threshold of *P* < 0.05. Normality assumptions were verified through Shapiro-Wilk testing before group comparisons. Parametric analysis of normally distributed continuous variables was performed using independent two-sample *t*-tests, while non-normally distributed variables were assessed via the nonparametric Wilcoxon rank-sum test. Categorical variables were analyzed using two-tailed Fisher’s exact tests to account for small sample sizes. Survival outcomes were visualized through Kaplan–Meier curves generated with the ‘ggplot2’ and ‘survminer’ packages, with between-group survival disparities quantified by log-rank testing.

## Result

### Analysis of transcriptomic data for gene expression profiling

**Figure 1:**
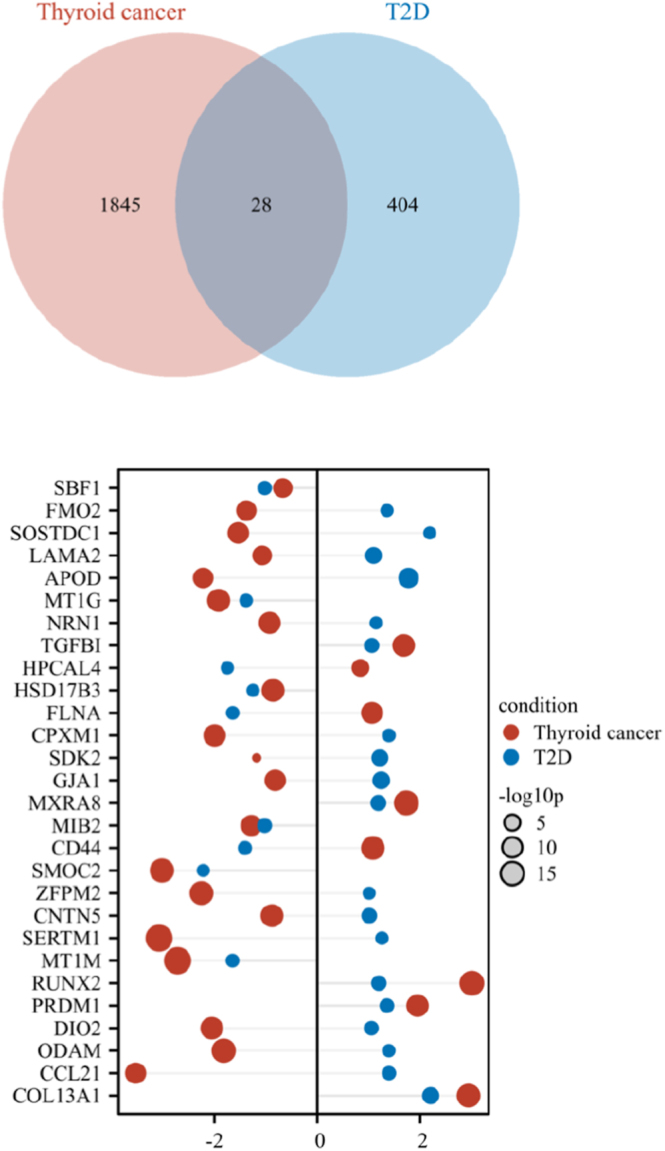
A summary of the transcriptome analysis. (A) Utilizing a Venn diagram, identify the common indicator genes between TC and type 2 diabetes mellitus (T2D). (B) Common gene bubble graph with associated log10 AP-value (log10 adjusted *P*-value) and log2 fold change.

According to our inclusion and exclusion criteria, the final statistical data for T2D and TC are shown in Table 1. This includes the following information: GEO accession number, data source, number of control and case specimens, and study year. To determine gene-level contributions to T2D and TC comorbidity, we processed RNA-Seq data obtained from NCBI. In addition, we processed the transcriptome sequencing data in both raw read counts and fragments per kilobase per million mapped reads (FPKM) formats, utilizing the count data for DEG analyses. TC was analyzed using Affymetrix Human Genome U133 Plus 2.0 Array (GPL570), including GSE33630, GSE35570, and GSE60542 (*n* = 211, TC *n* = 110, normal thyroid (NT) *n* = 101). T2D (GSE86468) was analyzed using GPL18573 Illumina NextSeq 500 for RNA sequence extraction (*n* = 24, T2D *n* = 9, ND *n* = 15). The significance threshold for DEGs was set to abs (log2 FC) >1 and *P* < 0.05. Differential expression analysis conducted using the ‘DESeq2’ package in R identified 1,873 DEGs in TC and 432 in T2D, with 28 genes shared between the two conditions (Fig. 1A). Among these shared genes, eight were upregulated and 20 downregulated in TC, while 19 were upregulated and 9 downregulated in T2D (Fig. 1B).

**Table 1:** The information of datasets from the GEO database.

Accession number	Samples		Year
	Tumor	Normal	
GSE33630	49	45	2012
GSE35570	33	32	2015
GSE60542	28	24	2015
		**T2D**	**Normal**
GSE86468	9	15	2016

### Exploring pathways of co-expressed gene modules and enriched KEGG pathways

**Figure 2:**
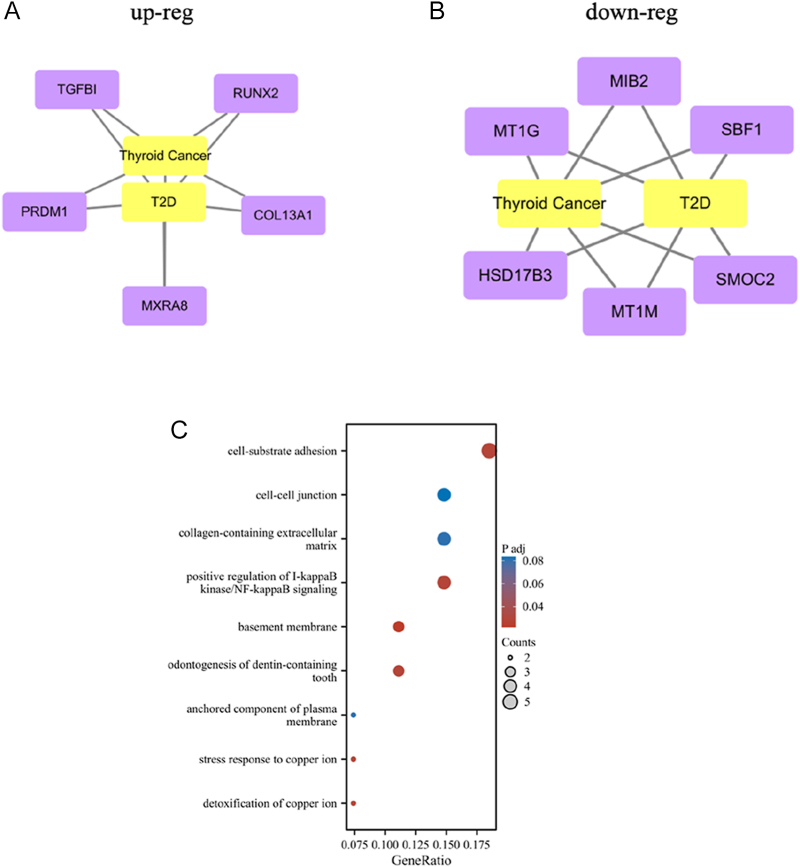
Analysis of co-expressed gene modules and enriched Kyoto Encyclopedia of Genes and Genomes (KEGG) pathways. (A and B) Genes that are up-regulated (up-reg) and down-regulated (down-reg) in type 2 diabetes (T2D) patients and TC patients are shown separately. (C) A bubble graph based on a transcriptome analysis illustrates nine major pathways associated with both diseases. The relevance degree of each pathway is shown by the log10 AP-value.

Using R program for identification and screening, we identified six downregulated genes shared by the two diseases: SBF1, MT1G, HSD17B3, MIB2, SMOC2, and MT1M. In addition, five upregulated genes were found: TGFBI, MXRA8, RUNX2, PRDM1, and COL13A (Fig. 2A). Based on these co-expressed genes, we performed KEGG analyses to investigate the commonly enriched pathways shared between TC and T2D. KEGG pathway enrichment was performed in R program using the ‘ggkegg’ package. Given that the adjusted *P*-value was less than 0.05, the pathways were ultimately ranked in ascending order based on the adjusted *P*-values. We ultimately identified nine signaling pathways linked to both diseases. Figure 2B illustrates these pathways associated with T2D and TC. Remarkably, the cell-substrate adhesion, cell–cell junctions, and collagen-containing extracellular matrix pathways play a crucial role in both diseases.

### Classification of hub proteins and submodules

**Figure 3:**
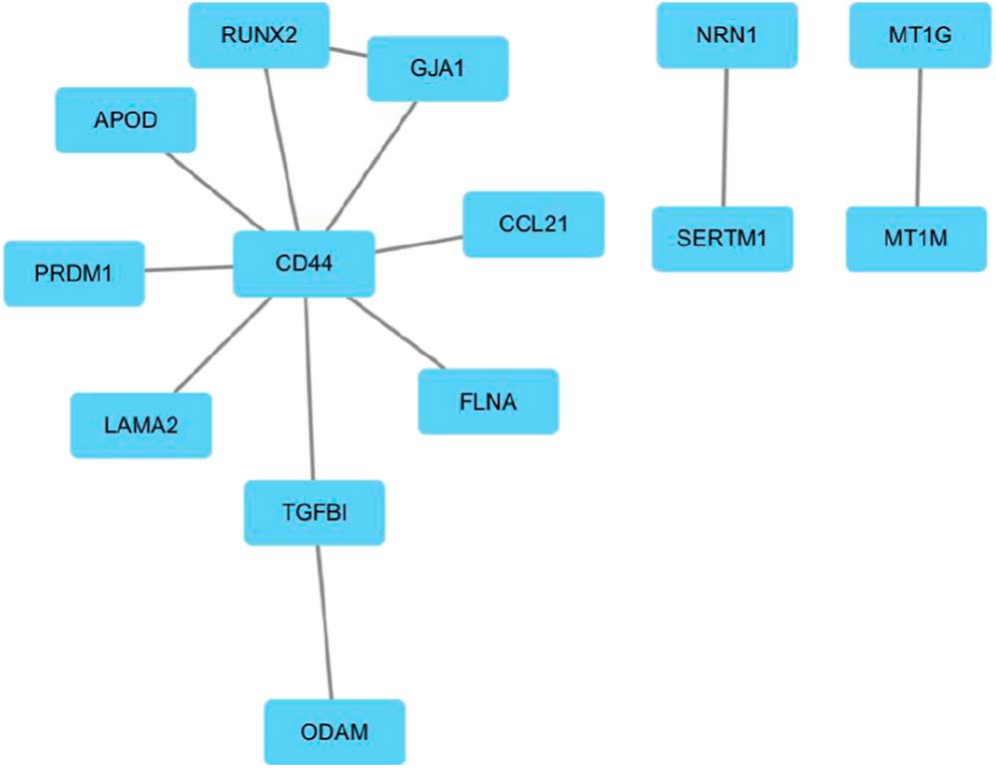
The figure describes the PPI network of DEGs between type 2 diabetes mellitus (T2D) and TC. Square-shaped nodes indicate DEGs, and edges denote node relations.

We constructed a PPI network based on the DEGs shared by TC and T2D using the STRING database. Figure 3 presents the construction and analysis of this network in Cytoscape. The resulting PPI network comprises 14 nodes and 12 edges, with CD44 positioned at the core, acting as a crucial connecting hub. These nodes are linked to protein subnetworks associated with TC and T2D. Moreover, the newly identified hub proteins demonstrate potential therapeutic value and merit further investigation to better understand their roles.

**Figure 4:**
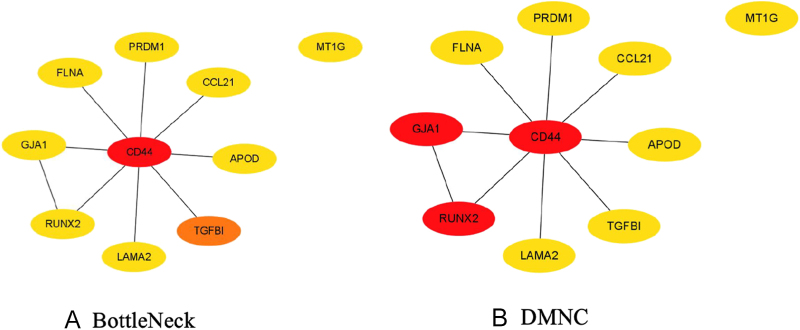
Cytoscape’s CytoHubba plugin was used to find hub genes in the PPI network. The latest versions of the BottleNeck and density of maximum neighborhood component (DMNC) CytoHubba plugins were employed to collect hub genes.

Hub protein identification was performed using both BottleNeck and DMNC centrality analyses (Fig. 4), providing orthogonal validation of key network regulators. These parameters, calculated through different algorithms, reflect the significance of nodes within the overall network from various perspectives. Using the BottleNeck algorithm, the top ten significant genes were identified and marked in red, orange, and yellow. Similarly, the DMNC algorithm was employed to mark the top ten hub genes in red, orange, and yellow, allowing for the calculation of overlapping genes between the two methods. To analyze the interconnectivity and closeness of the hub genes, we constructed two submodule networks. A higher degree of connectivity indicates that these genes rank within the top 10% in terms of interconnections. Based on both algorithms, we identified the top ten DEGs as the most significant: CD44, FLNA, PRDM1, CCL21, APOD, TGFBI, LAMA2, RUNX2, GJA1, and MT1G. Remarkably, independent BottleNeck and DMNC centrality analyses unanimously identified ten overlapping hub genes, establishing them as prime candidates for both diagnostic biomarkers and multi-target therapeutic development in these comorbid conditions.

### Construction of the risk prediction model and screening of prognostic core DEGs

**Figure 5:**
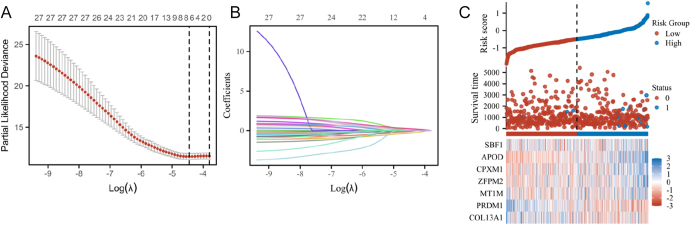
Screening and risk assessment of DEGs using LASSO. (A) Correlation between log(λ) and the mean-squared error in the least absolute shrinkage and selection operator (LASSO) Cox regression model. (B) Correlation between log(λ) and the coefficients of relevance in the LASSO Cox regression model. (C) Using an adequate risk score threshold, TC patients were split into high-risk and low-risk categories.

**Figure 6:**
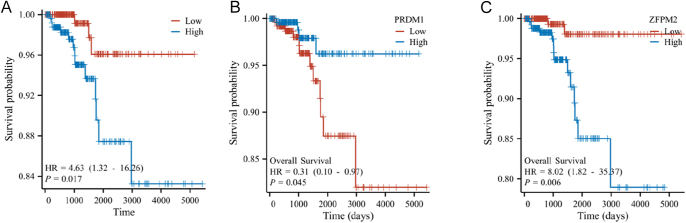
Kaplan–Meier (KM) plots demonstrated survival differences among TC patients across various categories. (A) KM plots showed TC patients of high-risk and low-risk. (B) KM plots showed TC patients with high and low expression of PRDM1. (C) KM plots showed TC patients with high and low expression of ZEPM2.

We conducted univariate Cox analysis to examine the relationship between these DEGs and overall survival (OS). Subsequently, LASSO-penalized multivariate Cox modeling was performed over 100 simulation repetitions, resulting in the construction of an optimal model comprising seven coefficients (Fig. 6A and B). Among these, PRDM1, COL13A1, and MT1M were identified as protective factors, whereas SBF1, APOD, CPXM1, and ZEPM2 were classified as risk factors. The predicted high- and low-risk groups from the model, alongside actual events and corresponding gene expression patterns, are presented in Fig. 5C.

Using the LASSO model, we stratified TC patients into high- and low-risk groups and evaluated their association with OS. As illustrated in Fig. 6A, the high-risk group exhibited a markedly worse prognosis compared to the low-risk group (*P* = 0.017). The gene expression levels of the seven genes were categorized into high- and low-expression groups based on the median expression values across all samples, and the associated survival differences were displayed in Kaplan–Meier plots. The results indicated that TC patients with high PRDM1 expression had a better prognosis (HR = 0.31, 95% CI = 0.10–0.97, *P* = 0.045) (Fig. 6B). Conversely, patients with high ZEPM2 expression exhibited a poorer prognosis (HR = 8.02, 95% CI = 1.82–35.37, *P* = 0.006) (Fig. 6C). These two genes demonstrated significant correlations with the OS of TC patients, underscoring the reliability of our risk prediction model.

### Assessing predictive values and stability of the IRS model

To systematically evaluate associations between potential targets and the clinicopathological characteristics of TC, we constructed Table 2. The clinicopathological characteristics included pathological T stage, N stage, M stage, tumor stage, and the presence of extrathyroidal extension. In addition, we analyzed the relationship between PRDM1 and ZFPM2 expression levels (high-expression vs low-expression groups) and OS. The results revealed that pathological stages T3 and T4, compared to T1 and T2, were significant prognostic risk factors in univariate analysis (HR = 3.002, 95% CI = 1.041–8.656, *P* = 0.042), underscoring that tumors larger than 4 cm and invasion of the surrounding structures are independent predictors of poor outcomes in TC. Although no significant association was found between prognosis and N or M stages, patients with M1 disease (*n* = 9) demonstrated a trend toward worse survival outcomes (*P* = 0.066). Pathological and clinical analyses indicated that advanced-stage TC (stages III and IV) significantly increased the risk of cancer-related mortality (HR = 7.263, 95% CI = 2.337–22.573, *P* < 0.001), a result that remained consistent in multivariate analysis (HR = 5.783, 95% CI = 1.538–21.749, *P* = 0.009). Of note, PRDM1 was identified as a protective factor in univariate analysis (HR = 0.31, 95% CI = 0.10–0.97, *P* = 0.045). In contrast, ZFPM2 was shown to be a significant risk factor for poor prognosis in both univariate (HR = 8.02, 95% CI = 1.82–35.37, *P* = 0.006) and multivariate analyses (HR = 11.532, 95% CI = 2.564–51.856, *P* = 0.001).

**Table 2:** Univariate and multivariate Cox regression analysis of associated genes and the clinicopathological characteristics.

Characteristics	Total (*n*)	Univariate analysis		Multivariate analysis	
		Hazard ratio (95% CI)	*P* value	Hazard ratio (95% CI)	*P* value
Pathologic T stage	510				
T1&T2	312				
T3&T4	198	3.002 (1.041–8.656)	**0.042**	1.049 (0.300–3.674)	0.940
Pathologic N stage	462				
N0	229				
N1	233	1.405 (0.458–4.308)	0.552		
Pathologic M stage	295				
M0	286				
M1	9	4.258 (0.909–19.952)	0.066		
Pathologic stage	510				
Stage I & stage II	340				
Stage III & stage IV	170	7.263 (2.337–22.573)	**<0.001**	5.783 (1.538–21.749)	**0.009**
Extrathyroidal extension	494				
No	340				
Yes	154	2.274 (0.843–6.130)	0.105		
PRDM1	512				
Low	256				
High	256	0.314 (0.101–0.974)	**0.045**	0.347 (0.105–1.143)	0.082
ZFPM2	512				
Low	256				
High	256	8.016 (1.817–35.365)	**0.006**	11.532 (2.564–51.856)	**0.001**

Bold indicates statistical significance, P < 0.05.

## Discussion

TC shows global prevalence with strong female predominance, and diabetes has been identified as a potential risk factor for increasing its incidence in females (34). As a result, some experts recommend regular thyroid screenings for patients with T2D (35). A study by Dong *et al.* further corroborated this, revealing that individuals with any form of diabetes face a higher risk of developing TC compared to ND individuals. This positive association between T2D and TC risk was consistent in both men and women (8). Diabetes and TC share overlapping symptoms, with evidence suggesting potential mechanisms linking the two conditions. These mechanisms include insulin resistance, obesity, hyperglycemia, hyperlipidemia, elevated thyroid-stimulating hormone (TSH) levels, the use of antidiabetic medications such as insulin and sulfonylureas, and vitamin D deficiency (36). Significantly, hyperglycemia can directly or indirectly facilitate cancer cell proliferation, migration, invasion, and immune evasion through multiple pathways (37).

Although diabetes and TC have distinct etiologies, their coexistence may intensify symptom severity. Insulin resistance and its associated metabolic disturbances, including hyperinsulinemia, estrogen-dependent signaling, and chronic autoimmune thyroiditis, have been implicated in an increased risk of TC (38). Various cytokines, such as IGF-1 levels, and PI3K, further contribute to this elevated risk. Strikingly, TC exhibits a higher density of IGF-1 receptor and insulin receptor isoform C, underscoring their biological significance in cancer progression (39). Emerging evidence links glucagon-like peptide-1 receptor (GLP-1R) agonists to an increased risk of TC (21). The impact of T2D medications on TC progression is complex, with some drugs demonstrating protective effects while others elevate risk. For instance, metformin, a widely used glucose-lowering agent in T2D, has been shown to inhibit TC cell proliferation and induce apoptosis through pathways such as AMPK, TSC2/mTOR, insulin receptor substrate-1, IGF-1R, and PI3K/AKT (40). In addition, metformin acts via AMPK-independent mechanisms, such as suppressing the unfolded protein response (UPR), thereby promoting apoptosis, inhibiting angiogenesis, and exerting toxic effects on cancer stem cells. Retrospective and large-scale prospective studies have consistently demonstrated a significant reduction in TC incidence among metformin users (41). In contrast, another study revealed that GLP-1 receptor agonists, including semaglutide, liraglutide, and exenatide, are associated with an increased risk of all TCs, particularly medullary TC, with the risk being most pronounced during 1–3 years of treatment (42). The intricate relationship between T2D and TC highlights the challenges in unraveling their shared mechanisms. While T2D is recognized as a potential promoter of TC development, the underlying pathways remain poorly understood, hindering hypothesis-driven research. To address this, we sought to explore the pathways and interactions between these two diseases using bioinformatics analysis.

We employed advanced computational biology approaches to systematically characterize dysfunctional gene networks and signaling pathways, with the specific objective of identifying molecular mediators underlying the T2D-TC disease interaction. GEO RNA-seq datasets from public databases were processed to perform an in-depth analysis of how these two diseases interact. The RNA-seq dataset for T2D was derived from T2D patients and ND individuals, while the thyroid dataset was obtained from healthy controls and case groups. Our findings identified a set of DEGs shared by T2D and TC, which were subsequently used to construct a predictive model. These DEGs provided insights into potential molecular targets and key signaling pathways involved in disease progression. We utilized the STRING database to examine PPIs linked to the shared DEGs and constructed a PPI network using topological parameters. To enhance the reliability of node importance predictions, we employed the BottleNeck and DMNC algorithms for validation (43, 44). The PPI network analysis underscored critical protein interactions potentially implicated in the pathogenesis of both diseases. Based on connectivity rankings, we highlighted the top ten interactive proteins, with CD44, TGFBI, GJA1, and RUNX2 emerging as prominent hub proteins in this study.

The cleavage of CD44 protein is known to enhance cAMP response element-binding protein phosphorylation, thereby sustaining the proliferation of TC cells (45). Previous studies have also highlighted a significant association between CD44 and T2D (46), suggesting that CD44 may serve as a crucial mediator in the molecular interactions between T2D and TC. Similarly, RUNX2 has been shown to promote aortic fibrosis and stiffness, contributing to cardiovascular complications in T2D patients (47). Moreover, RUNX2 plays a key regulatory role in epithelial-to-mesenchymal transition and invasion in TC, emphasizing its importance in both diseases (48). On a similar note, TGFBI, an extracellular matrix protein, is overexpressed in various cancers, including TC (49, 50). It has also been identified as a diabetes risk gene in human genetic studies, although its specific involvement in the metabolic disturbances of T2D remains unclear (51). The connexin 43-encoding gene GJA1 is overexpressed in cervical, colorectal, and breast cancers, exhibiting established prognostic value in these malignancies (52, 53, 54, 55). However, its role in TC is yet to be thoroughly explored, representing a potential area for future investigation. Critically, connexin 43 is also recognized as a therapeutic target for addressing inflammation in secondary complications of diabetic nephropathy and retinopathy. PPI in Fig. 4 confirms the importance of several proteins in both TC and T2D, as documented in previous studies. We posit that the remaining proteins in this analysis hold substantial potential for further research and may unveil critical insights into the molecular mechanisms linking these two diseases.

Pathway analysis is a widely utilized tool in life science research, designed to extract meaningful insights from high-throughput biological data. In this study, nearly all identified pathways are associated with ‘cell adhesion’, ‘inflammatory signaling regulation’, and ‘copper ion metabolism’ (Fig. 2). Pathways such as ‘cell-substrate adhesion’, ‘cell–cell junctions’, ‘collagen-containing extracellular matrix pathways’, ‘basement membrane’, and ‘anchored components of the plasma membrane’ are closely tied to ‘cell adhesion’ mechanisms. Cell adhesion plays a dual role: it physically anchors cancer cells and serves as a critical mediator of signaling between the extracellular microenvironment and cells. Of note, cell adhesion molecules are pivotal in processes such as the loss of intercellular adhesion and anchorage-independent growth, which promote carcinogenesis, tumor progression, cell proliferation, and metastasis (56). Numerous studies have shown that mutations in genes related to cell adhesion are strongly linked to the aggressiveness of TC (57, 58). Emerging evidence also supports the role of ‘intercellular junctions (IJs)’ in mediating cell–cell contact during the pathogenesis of T2D. IJs influence endocrine function, pancreatic activity, intestinal barrier integrity, and renal function, all of which contribute to the onset and progression of T2D-related metabolic dysfunctions (59). The ‘I-kappaB kinase/NF-kappaB signaling pathway’ is one of the most critical pathways regulating inflammation and stress responses. It not only plays a key role in physiological processes such as inflammation regulation, stress response, and cell survival, but also serves as a major driver of cancer initiation and progression (60). Studies by Yin *et al.* have demonstrated a positive correlation between plasma copper concentration and impaired glucose regulation as well as T2D (61). Furthermore, treatment with copper chelators has been shown to reduce insulin resistance and improve glucose intolerance in diabetic mice, suggesting that copper ions play a role in the development of T2D (62). This highlights copper ions as potential therapeutic targets for diabetes (63). Extensive research has also reported significantly elevated serum and tumor copper levels in various malignancies, with high copper levels directly linked to cancer progression (64). This is likely due to a combination of factors such as increased metabolic activity within tumors, mitochondrial mutations, cytokine production, and inflammation, which collectively enhance oxidative stress in the tumor microenvironment. Consequently, recent studies suggest that cancer cells’ characteristic copper accumulation and associated oxidative stress may present promising opportunities for selective cancer therapies (65). These findings suggest that the identified pathways play critical roles in the metabolic dysregulation associated with both TC and T2D, contributing to their onset and progression.

In our predictive model, we identified SBF1, APOD, CPXM1, and ZEPM2 as risk factors for TC, while MT1M, PRDM1, and COL13A1 were determined to be protective factors (Fig. 5). SBF1 has been reported as a potential biomarker for gemcitabine sensitivity in head and neck squamous cell carcinoma cell lines (65). In addition, studies have shown that SBF1 plays a key role in the development of chronic myeloid leukemia (66, 67). In a multicenter retrospective study, Wang *et al.* found that high APOD expression is significantly associated with poor clinical outcomes, reduced microsatellite instability, lower tumor mutational burden, altered tumor microenvironment, and reduced response to immunotherapy (68). Furthermore, APOD was suggested as a potential therapeutic target for cervical cancer in an immune prediction model (69). APOD also demonstrates prognostic value in colorectal, thyroid, pancreatic, liver, and prostate cancers (65). T2D patients exhibit apolipoprotein disturbances (including APOD deficiency), which correlate with dyslipidemia and complication risk (65). CPXM1, another gene identified in our model, is upregulated in gastric cancer, correlating with poor prognosis, gender, and tumor stage. Functional enrichment analysis suggests CPXM1 influences gastric cancer progression through the PI3K-AKT and TGF-β pathways. Furthermore, CPXM1 expression is positively correlated with M2 macrophages (70). CPXM1’s role in local tumor immune infiltration has been confirmed in head and neck squamous cell carcinoma, where an immune-related gene prognosis model incorporating CPXM1 indicates that patients with high CPXM1 expression exhibit increased immune suppression, a more aggressive phenotype, and reduced benefit from immune checkpoint inhibitors (71). CPXM1 has also been implicated in ovarian, cervical, and bladder cancers (72, 73, 74).

In contrast, ZEPM2 has limited cancer-related research. It is an autosomal dominant gene involved in GATA4 and GATA6 protein binding. Long non-coding RNA ZFPM2-AS1 and the ZFPM2 gene are associated with hepatocellular carcinoma prognosis (75), but its role in TC requires further investigation. Crucially, MT1M is downregulated in TC and is an independent risk factor for lymph node metastasis in PTC. *In vitro* studies show that MT1M upregulation significantly inhibits colony formation, proliferation, migration, and invasion of PTC cells (76). MT1M’s tumor-suppressive effects have also been confirmed in hepatocellular carcinoma (77), potentially due to promoter methylation of MT1M and MT1G (78). MT1M also suppresses esophageal cancer progression by inhibiting epithelial–mesenchymal transition and the SOD1/PI3K axis (79). Notably, MT1 is a negative regulator of insulin secretion, with decreased MT1 associated with β-cell compensation in obesity, while increased MT1 correlates with β-cell failure in T2D (80). Besides, PRDM1 functions as a tumor suppressor in B-cell and T-cell lymphomas, silencing stem cell-related genes and inhibiting the proliferation of human colorectal tumor organoids (81). It has also been identified as a tumor suppressor in non-Hodgkin lymphoma and a susceptibility locus for systemic lupus erythematosus (82, 83). COL13A1, produced by urothelial carcinoma cells, supports tumor invasion and may serve as a diagnostic and prognostic biomarker for bladder cancer (84). In addition, COL13A1 expression is elevated in bone metastatic prostate cancer compared to localized disease (85). Although there are no studies on COL13A1 in TC, its potential role merits further exploration. Collectively, these seven key genes hold significant potential for research into both TC and T2D, providing new avenues for future investigations.

It is important to note that our study has certain limitations. The GEO datasets used in our analysis were limited by relatively small sample sizes and lacked comprehensive clinical annotations, including demographic variables (e.g., age, gender, and race), treatment histories, and lifestyle factors, which may influence the observed molecular patterns. While our findings provide valuable insights, future studies with larger, well-annotated cohorts and experimental validation are needed to confirm these results and further explore their biological and clinical significance.

## Conclusion

In summary, our study integrates multi-omics data to systematically unravel the shared genetic architecture of T2D and TC. We identified seven key genes underpinning this comorbidity and constructed a robust prognostic model capable of stratifying TC patients into distinct risk groups. Further gene screening revealed that PRDM1 and ZFPM2 act as protective and harmful factors, respectively, and hold potential as targets for future exploration. These results decode novel metabolic-epigenetic networks in endocrine malignancies while revealing druggable targets with specific therapeutic potential for diabetes-associated TCs.

## Declaration of interest

All the authors declare that the work was conducted in the absence of any commercial or financial relationships that could be construed as a potential conflict of interest.

## Funding

This work was supported by grants from Wenzhou Medical University Affiliated First Hospital Doctoral Research Initiation Fund (2022QD048).

## Author contribution statement

Jiahui Qi helped in conceptualization, data curation, formal analysis, methodology, and manuscript writing. Chuanzhi Chen was responsible for investigation, software, visualization, validation, and manuscript writing. Feng Zhu contributed to text refinement, reference verification, and figure formatting. Chuankai Chen helped in literature review and manuscript preparation. Yue Wang was responsible for conceptualization, overall supervision, critical revision of the manuscript, and final approval of the manuscript.

## Statement of ethics

The data used in this study were entirely sourced from publicly accessible databases, particularly the Gene Expression Omnibus (GEO) database (available at: https://www.ncbi.nlm.nih.gov/geo/). The use of data complied with the access terms and licensing agreements of these databases.
